# Differential microRNA expression in breast cancer with different onset age

**DOI:** 10.1371/journal.pone.0191195

**Published:** 2018-01-11

**Authors:** Hsiu-Pei Tsai, Shiang-Fu Huang, Chien-Fan Li, Huei-Tzu Chien, Shin-Cheh Chen

**Affiliations:** 1 Graduate Institute of Clinical Medical Sciences, Chang Gung University, Gueishan, Taoyuan, Taiwan; 2 Department of General Surgery, Chang Gung Memorial Hospital, Gueishan, Taoyuan, Taiwan; 3 Department of Public Health, Chang Gung University, Gueishan, Taoyuan, Taiwan; 4 Department of Otolaryngology, Head and Neck Surgery, Chang Gung Memorial Hospital, Gueishan, Taoyuan, Taiwan; University of North Carolina at Chapel Hill School of Medicine, UNITED STATES

## Abstract

**Purpose:**

The lower breast cancer incidence in Asian populations compared with Western populations has been speculated to be caused by environmental and genetic variation. Early-onset breast cancer occupies a considerable proportion of breast cancers in Asian populations, but the reason for this is unclear. We aimed to examine miRNA expression profiles in different age-onset groups and pathological subtypes in Asian breast cancer.

**Methods:**

At the first stage, 10 samples (tumor: n = 6, normal tissue: n = 4) were analyzed with an Agilent microRNA 470 probe microarray. Candidate miRNAs with expression levels that were significantly altered in breast cancer samples or selected from a literature review were further validated by quantitative real-time PCR (qPCR) of 145 breast cancer samples at the second stage of the process. Correlations between clinicopathological parameters of breast cancer patients from different age groups and candidate miRNA expression were elucidated.

**Results:**

In the present study, the tumor subtypes were significantly different in each age group, and an onset age below 40 had poor disease-free and overall survival rates. For all breast cancer patients, miR-335 and miR-145 were down-regulated, and miR-21, miR-200a, miR-200c, and miR-141 were up-regulated. In very young patients (age < 35 y/o), the expression of 3 and 8 specific miRNAs were up- and down-regulated, respectively. In young patients (36–40 y/o), 3 and 3 specific miRNAs were up- and down-regulated, respectively. miR-532-5p was up-regulated in triple-negative breast cancer.

**Conclusions:**

Differential miRNA expressions between normal and tumor tissues were observed in different age groups and tumor subtypes. Evolutionarily conserved miRNA clusters, which initiate malignancy transformation, were up-regulated in the breast cancers of very young patients. None of the significantly altered miRNAs were observed in postmenopausal patients.

## Introduction

The median age of breast cancer diagnosis in Taiwan (45–49 years) is lower than that of Western countries (70–74 years) [1]. In Taiwan, approximately 7% of all breast cancers were diagnosed in patients younger than 40 years of age, and less than 4% were younger than 30 years of age [2]. The percentage of young breast cancers is much higher than that in Western countries. A higher prevalence of luminal A subtype is observed in the young aged breast cancer in Taiwan. Genetic background as well as environmental factors might interact and contribute to the specific trend in breast cancer in Taiwan [3]. It has long been observed that young women with breast cancer are more likely to suffer recurrence and death than older women, even if diagnosed early and treated intensively [4]. The poor prognosis of breast cancers occurring in young women needs to be paid more attention.

Although the government launched paid bi-annular mammography screening in 2002 for women between 50 and 69 years and in 2009 for women over 45 years, or 40 years with a high risk of breast cancer, there has been no screening project or official suggestion for young women. In addition, differences in the molecular subtypes of tumors and treatment modality may affect the prognosis and survival among different age groups. As can be observed in the literature, young women share aggressive histopathological tumor features. Quantitative measurements show lower estrogen receptor (ER) mRNA expression, higher HER2 mRNA expression, and higher prevalence of triple-negative breast cancer among tumors arising in young women [5]. After controlling for different classical prognostic factors, most studies have shown that young age remains a powerful predictor of poor outcome. Gene expression profiling can explore clues as to the causes of this age effect at the molecular level. Beyond traditional clinicopathological parameters, BRCA1, apoptosis, hypoxia, stem cell biology, histone deacetylase, the mTOR/rapamycin pathway, and oncogenic signaling pathways such as p53, PTEN, MAP kinase, AKT, Myc, β-catenin, and E2F hold prognostic and therapeutic implications in breast cancer treatment. However, the biological pathways contributing to pathogenesis in young breast cancer have not been fully explored. Studies have shown that hereditary breast cancer (e.g., BRCA1 and 2 mutations) are frequent in breast cancers in young women, but the prevalence of high penetrance gene mutations was lower in Asian populations [6, 7]. Biological factors driving breast cancer susceptibility in young Asian women deserve to be investigated.

MicroRNAs (miRNAs), small endogenous noncoding functional RNAs, have been shown to be involved in mammary gland development, proliferation and breast cancer [8]. Studies have demonstrated that expression of miRNAs is associated with several types of cancers, tumor invasiveness and metastatic potential [9, 10]. The up- or down-regulation of tissue-specific miRNAs has been recognized as a major regulatory gatekeeper of signal transduction pathways and other biological pathways. MicroRNAs have been suggested to be new laboratory biomarkers and therapeutic targets [11].

The aim of the current study was to identify miRNA expression profiles in different age groups and pathological subtypes. By investigating the biological nature of breast cancers with different onset ages, we explored the possible pathogenic pathways leading to early-onset breast cancer and unique clinicopathological expression in Asian women.

## Materials and methods

### Experimental design

**Fig 1:**
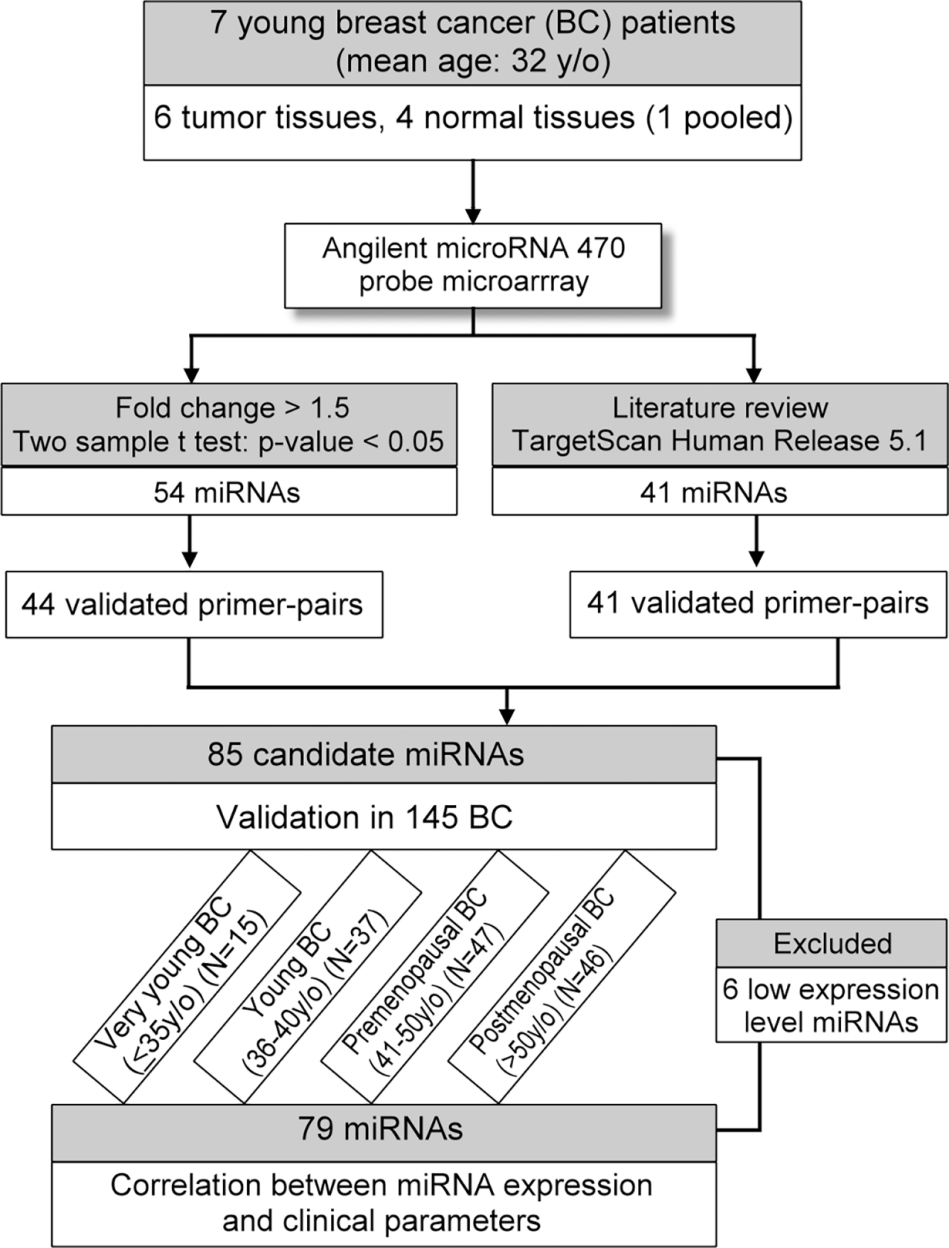
Flow chart diagram of the study design.

This study was approved by the Chang Gung Medical Foundation Institutional Review Board (98-0776B). The two-stage experimental design is illustrated by a flow chart (Fig 1). First, the candidate miRNAs were selected from miRNA microarray analysis or literature review. At the second stage, a total of 85 miRNAs were validated in 145 breast tumor tissue samples (only 140 samples with paired adjacent normal tissues) from different age groups of breast cancer patients by quantitative real-time PCR analysis (qPCR), and the correlation between differential expression miRNAs and clinicopathological parameters was investigated.

### Patients, clinical diagnosis, and tissue specimen processing

Breast cancer patients who received curative mastectomies during 2001 and 2009 at the Chang Gung Memorial Hospital were selected for this study. We excluded patients who underwent neo-adjuvant chemotherapy or carried recurrent/metastatic diseases. We focused on those for whom pathological features such as invasive ductal carcinoma and paired tumor and normal tissues were available. All patients were questioned of their last menstruation period at outpatient department when visiting breast problems to confirm their pregnancy status. There was no pregnancy-associated breast cancer in this study. Patients were selected randomly without designated referent and being grouped according to their age. Patients who participated in the IRB-approved study signed an informed consent form. For each case, tumor and normal breast tissue samples were surgically dissected into small pieces, frozen immediately in liquid nitrogen and stored at -80°C until processing. Normal tissue was obtained as far as possible from tumor in the affected breast. For breast lumpectomy, the distance between tumor and normal tissue was at least 1cm. Routinely, we kept a free margin greater than 1.5cm when performing partial mastectomy. In the first process, pathological reports such as in situ ductal carcinoma was not excluded to obtain a broad range of miRNAs. In the second process, the selection was limited to invasive ductal carcinomas for a more specific analysis for the majority of breast cancers. The following clinicopathological variables of patients were collected for univariate and multivariate analyses: patient's age at diagnosis; tumor size; axillary lymph node status; UICC stage; ER, progesterone receptor (PR), and HER2/neu status; molecular subtypes; disease free and overall survival duration up to the last follow up date of December 31, 2016.

### Measurement of ER, PR, and HER2/neu

Tumor grading was performed according to the Eills and Elston grading system [12]. For immunohistochemistry (IHC) staining, the specimen was obtained with fixation time less than one hour to avoid false negative result. IHC staining for ER, PR and HER2/neu was carried according to the Envision method as a standard procedure for clinical purpose [13]. The tissue samples were classified as positive for ER and PR when more than 1% of the tumor cells showed positive staining. The DAKO-scoring system for HER2/neu was defined as negative when the score was zero, 1+, or 2+, and positive when a score of 3+ was given [13]. The interpretation of ER, PR, HER2/neu results followed the recommended guideline of the American Society of Clinical Oncology/College of American Pathologists.

### RNA extraction and miRNA microarray

Total RNA was extracted from tissues using TRIzol (Invitrogen, Calsbad, CA) according to the manufacturer's instructions. The quality and concentration of RNA was evaluated using a NanoDrop ND-1000 spectrophotometer. We constructed 10 sample (tumor: n = 6, normal tissue: n = 4) analyses by an Agilent microRNA 470 probe microarray (Agilent, Santa Clara, CA). miRNA nomenclature was adopted according to the miRBase sequence database 9.0. A total of 0.5 μg of total RNA was processed, labeled and hybridized to array chips according to the manufacturer's protocol. The array images were analyzed in the Partek Genomic Suite (version 6.4) for statistical analysis and data mining. Average values of the replicate spots of each miRNA were background subtracted and normalized, then subjected to further analysis. Normalization was performed with a per-chip median normalization method and the median array. Differentially expressed miRNAs were identified using the *t*-test procedure in significance analysis of the microarrays. miRNAs for which the fold-change was > 1.5 and the *t*-test *p* value was < 0.05 between tumor and normal breast tissues were selected for further qPCR analysis. miRNAs that were not expressed with a significant fold change but had been reported in the literature to be associated with breast cancer, or those with gene targets known to be critical for breast cancer, were also selected for qPCR validation.

### Reverse transcription (RT)

The RT reaction was performed using total RNA and random primers. The 22-μL volume RT reaction mixtures contained 1 μg of total RNA (0.5 μg/μL), 2 μL of random primers, and 9 μL of DEPC-treated water. The mixture was heated at 70°C for 5 minutes and quick-chilled on ice. The contents of the tube were collected by centrifugation, and 4 μL of dNTP (500 μM), 4 μL of 5 × first-strand buffer, and 1 μL of M-MLV (200 U) were added. The RT reaction was performed as follows: 16°C for 30 minutes followed by 50 cycles at 20°C for 30 seconds, 42°C for 30 seconds and 50°C for 1 second, then 70°C for 10 minutes to complete the procedure. The RT products were diluted 200-fold before using for miRNA qPCR.

### Quantitative real-time PCR analysis (qPCR)

Optimal primer concentrations were determined using optimization protocols from the Applied Biosystems SYBR Green PCR master mix manual. Reactions were performed in 10-μL volumes containing 0.5 μL of diluted RT product, 1× SYBR Green Master Mix (Applied Biosystems), miRNA-specific forward primer and universal reverse primer. The following PCR conditions were used: 95°C for 10 minutes, followed by 40 cycles at 95°C for 15 seconds and 64°C for 32 seconds. Samples were analyzed in duplicate. Each amplification reaction was checked for nonspecific PCR products by running the dissociation protocol. Two no-template controls (NTCs) were included on each experimental plate. All qPCR reactions were carried out on an ABI PRISM 7900 Sequence Detection System (Applied Biosystems). The mean threshold cycle (*C*_t_) of the duplicate analysis of each sample was calculated. Of the 85 miRNAs evaluated, 6 showed the expression level below the detection limit (*C*_t_ > 35) in more than 33.3% of samples, and were excluded from the analysis. For the remaining 79 miRNAs, raw *C*_t_ data were converted to 39-*C*_t_ normalized by global median normalization before further analysis [14]. The differential expressed miRNAs were selected based on fold change (> 2-fold), *t*-test (*p* < 0.05) and adjusted *p* value (*q* value < 0.05).

### Statistical analysis

The normalized data file was transposed and adjusted by Partek batch removal models (Partek Genomics Suite, version 6.3, St Louis, MO, USA) to eliminate technical and biological effects. Hierarchical clustering of miRNA microarray and qPCR data normalization were performed with the Partek Genomics Suite. Statistical analyses were performed with the SPSS statistical package version 13.0 (SPSS, Chicago, IL). Clinicopathological characteristics of different onset age groups were compared with chi-square test. *Student's t*-test was used to compare mean expression levels for significant differences between the tumor and normal breast tissues. The *p* values were adjusted by false discovery rate (FDR) for controlling multiple test errors. The disease-free and overall survival times were defined as the time interval from the date of surgery to the date of the first recurrence or death. The survival rate of 3, 5 and 10 years of different onset age groups were compared using ANOVA. Survival curves were constructed by the Kaplan-Meier method, and the curves were compared using the log-rank test. A two-sided *p* value < 0.05 was defined as statistically significant.

## Results

### Candidate miRNA selection from the microarray panel

At the first stage, 10 samples from 7 young breast cancer patients (tumor: n = 6, normal tissue: n = 4) were collected for RNA extraction and miRNA microarray analysis. Two patients donated paired samples, and one normal sample was produced by a mixture of 3 normal tissues to obtain an adequate amount of RNA. The mean age of the patients was 32.1 years (ranging from 28.6 to 35.1). Clinical stage, tumor subtype and immunohistochemical staining results of the patients are listed in Table 1. There were 54 miRNAs out of the Agilent 470 probe microarray that showed significant fold change between tumor and corresponding normal breast tissues (S1 Fig). Among them, 26 miRNAs were up-regulated and the other 28 miRNAs were down-regulated. Finally, the expression of a total of 44 miRNAs were analyzed successfully by quantitative real-time PCR (qPCR). Of these 44 miRNAs, there were 41 miRNAs that did not show significant fold change but had, or their target gene had, been reported in the literature or in TargetScan Human Release 5.1 (www.targetscan.org/) to be associated with breast cancer, and these miRNAs were also included for qPCR validation (Table 2).

**Table 1:** Clinicopathological parameters of subjects analyzed by miRNA microarray.

Number	Case ID	Tumor type	Age (years)	UICC Stage	ER status	PR status	HER2/neu
1	1 (T)	IDC	33.3	2A	negative	negative	positive
2	2 (N)	-	31.8	-	-	-	-
3	2 (T)	IDC	31.8	2A	positive	positive	negative
4	3 (N)	-	29.5	-	-	-	-
5	3 (T)	DCIS	29.5	0	positive	positive	negative
6	4 (T)	IDC	32.6	1	negative	negative	positive
7	5 (T)	DCIS	28.6	0	positive	positive	negative
8	6 (N)	-	35.1	-	-	-	-
9	8 (T)	IDC	33.7	2A	positive	positive	negative
10	P (N)	-	-	-	-	-	-

ID, identification; N, normal breast tissue; T, breast tumor; DCIS, ductal carcinoma in situ; IDC, invasive ductal carcinoma; P, pooled normal sample; ER, estrogen receptor; PR, progesterone receptor; HER2/neu, v-erb-b2 erythroblastic leukemia viral oncogene homolog 2 receptors; UICC, stage of breast tumor according to the international union for cancer staging criteria.

**Table 2:** List of selected candidate miRNAs for subsequent qPCR analysis.

MicroRNAs selected from microarray analysis (n = 44)									
miR-10b	miR-18a	miR-29a	miR-29c	miR-32*	miR-96	miR-98	miR-99a*	miR-101	miR-125b
miR-130a	miR-130b	miR-141	miR-143	miR-145	miR-148b	miR-149	miR-181c	miR-181d	miR-182
miR-183	miR-188-5p*	miR-196a	miR-199a-5p	miR-203	miR-301a	miR-320	miR-324-5p	miR-362-5p	miR-370
miR-374a	miR-375	miR-376a*	miR-429	miR-451*	miR-487b	miR-497	miR-513-5p	miR-532-5p	miR-565
miR-590-5p*	miR-625	miR-660	miR-671-5p						
MicroRNAs selected from the literature review (n = 41)									
let-7a	let-7c	let-7d	let-7f	let-7g	miR-9	miR-10a	miR-16	miR-17	miR-19a
miR-21	miR-25	miR-26a	miR-26b	miR-27a	miR-27b	miR-29b	miR-30a	miR-30b	miR-30c
miR-30d	miR-107	miR-124	miR-125a-5p	miR-126	miR-127-3p	miR-142-5p	miR-146a	miR-148a	miR-150
miR-152	miR-154	miR-155	miR-181b	miR-195	miR-200a	miR-200c	miR-205	miR-206	miR-335
miR-548c-5p									

* The 6 miRNAs with low expression levels (*C*_t_ > 35) that were excluded from clinicopathological analysis.

### Clinicopathological characteristics of investigated patients

**Fig 2:**
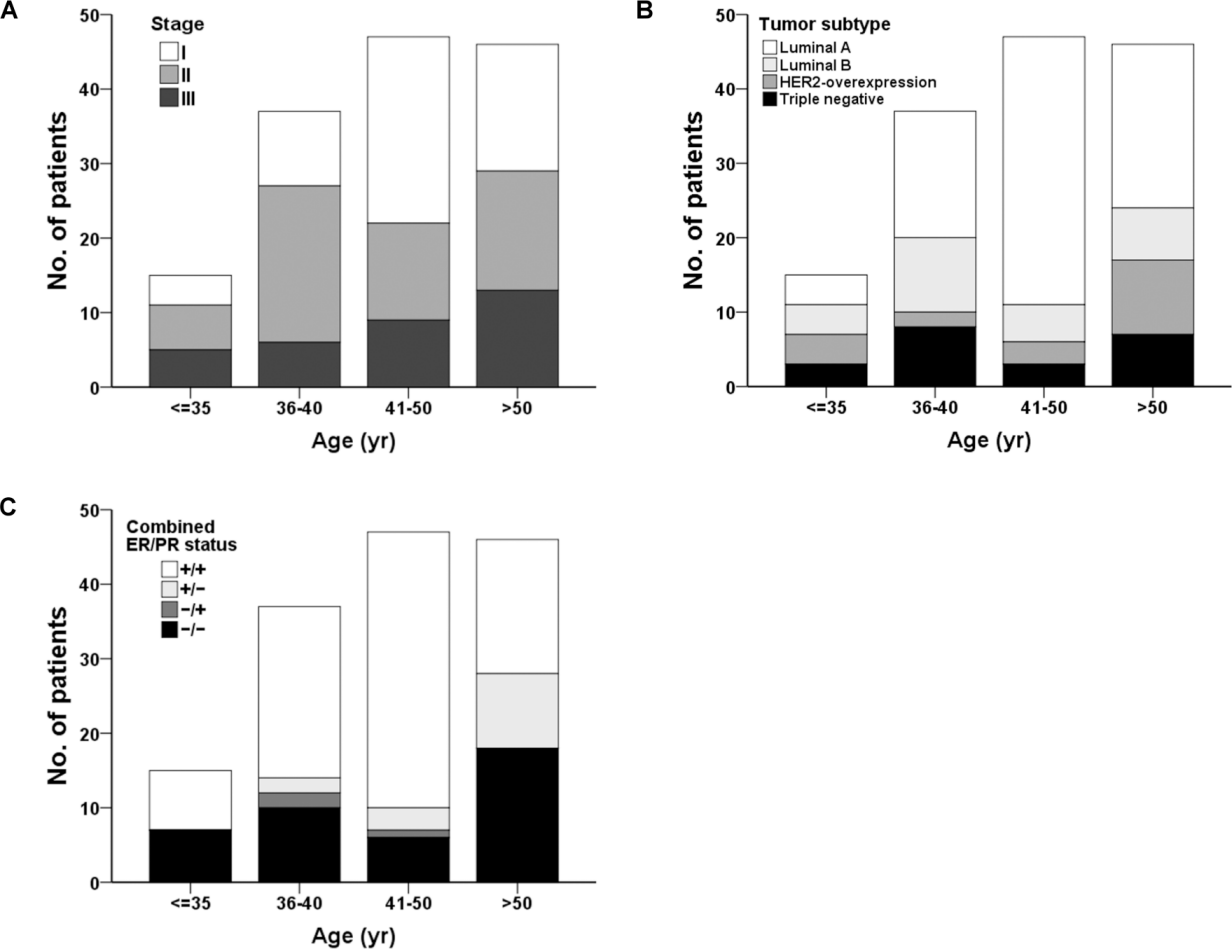
Stacked bar chart showing the distributions of clinicopathological status of breast cancer in different age groups. (A) tumor stage, (B) tumor subtype, and (C) combined ER, PR status in different age groups.

**Fig 3:**
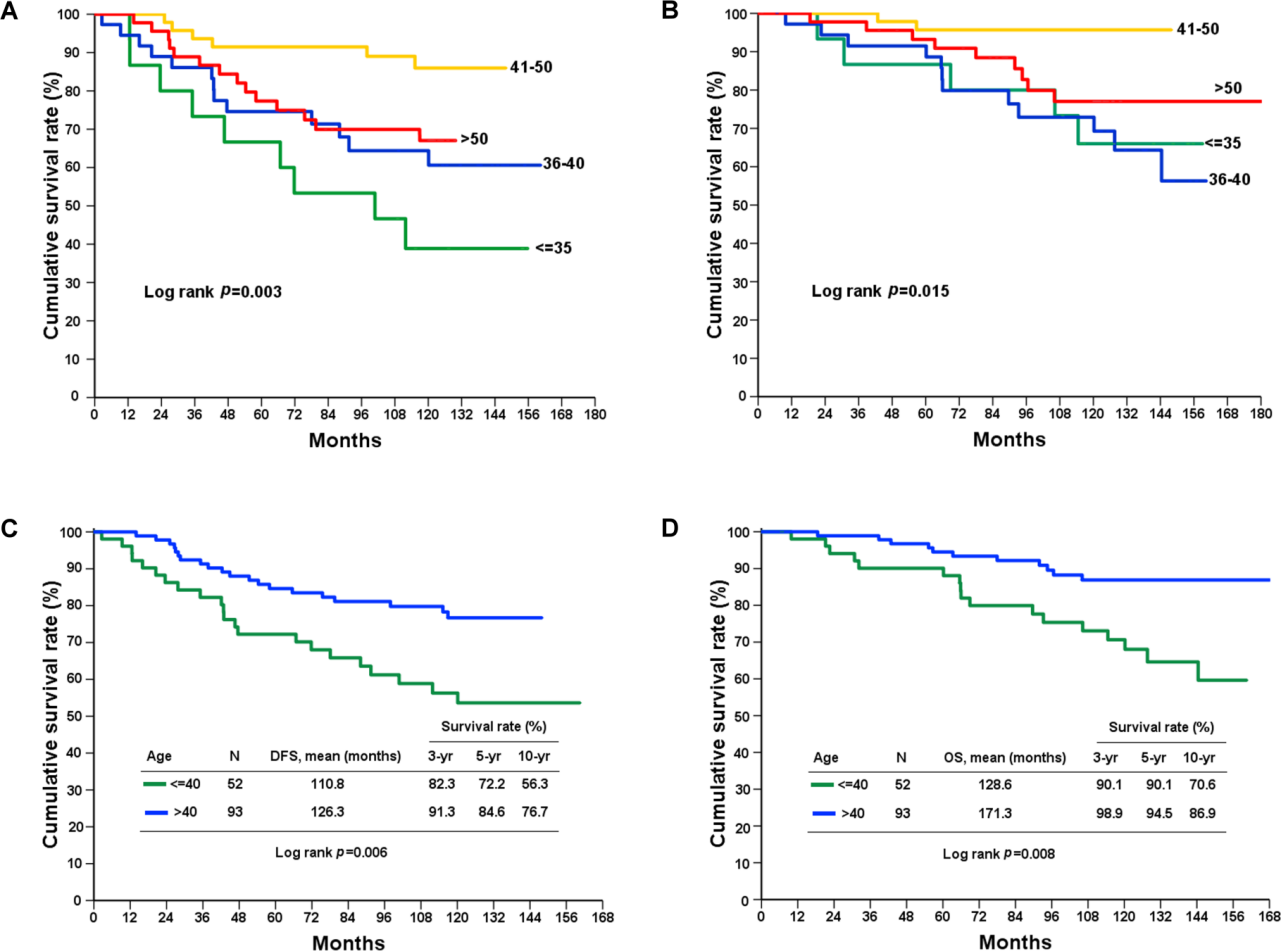
Kaplan-Meier survival curves for breast cancer patients. The young age group of patients had worse prognosis than other age groups in (A) disease-free (*p* = 0.003) and (B) overall survival (*p* = 0.015). Patients younger than 40 years had worse prognosis than the older age group in (C) disease-free (*p* = 0.006) and (D) overall survival (*p* = 0.008).

One hundred forty-five patients were included and divided into four onset age groups: very young (< 35 y/o), young (36–40 y/o), premenopausal (41–50 y/o) and postmenopausal (> 50 y/o). The median follow-up duration of patients was 119.7 months. The clinicopathological characteristics of each group of patients are listed in Table 3, and the disease-free and overall survival rates for all tumor stages are listed in Table 4. We found that the tumor molecular subtype, hormonal receptor status, disease free survival and overall survival rate had statistically significant differences between the four age groups (Figs 2 and 3), whereas tumor size, axillary LN status, cancer stage, and HER2/neu status did not exhibit significant differences between different ages. Patients had a lower hormone receptor-positive rate in the younger than 35 group. Additionally, patients older than 50 tended to have a higher PR receptor-negative rate. Among all patients, the very young group had the worst prognosis, whereas the premenopausal group had the best prognosis at the 10-year follow-up (Table 4). Age did not contribute to prognosis in stage I disease. However, in stage II and III breast cancer, patients younger than 40 had poor prognoses (Table 4).

**Table 3:** Clinicopathological characteristics of enrolled patients divided among four age groups.

	Age groups				
Characteristics	Very young	Young	Premenopausal	Postmenopausal	*P* value
	(< 35)	(36–40)	(41–50)	(>50)	
No. of patients	15	37	47	46	
Age at diagnosis (yrs)					
Mean (±SD)	33.4 (1.7)	38.0 (1.4)	45.8 (2.5)	59.2 (6.4)	< 0.0001*
Median (range)	34 (30–35)	38 (36–40)	46 (41–50)	58 (51–72)	
Tumor size (cm)					
Mean (±SD)	2.6 (1.9)	2.3 (1.0)	2.1 (1.5)	2.0 (1.0)	0.448
Median (range)	2.5 (0.9–9.0)	2.2 (0.2–4.3)	1.9 (0.1–7.3)	2.0 (0.6–5.4)	
Axillary LN status					
Positive	8 (53.3)	16 (43.2)	15 (31.9)	21 (45.7)	0.389
Negative	7 (46.7)	21 (56.8)	32 (68.1)	25 (54.3)	
TNM stage					
I	4 (26.7)	10 (27.0)	25 (53.2)	17 (37.0)	0.072
II	6 (40.0)	21 (56.8)	13 (27.7)	16 (34.8)	
III	5 (33.3)	6 (16.2)	9 (19.1)	13 (28.2)	
ER					
Positive	8 (53.3)	25 (67.6)	40 (85.1)	28 (60.9)	0.030*
Negative	7 (46.7)	12 (32.4)	7 (14.9)	18 (39.1)	
PR					
Positive	8 (53.3)	25 (67.6)	38 (80.9)	18 (39.1)	< 0.001*
Negative	7 (46.7)	12 (32.4)	9 (19.1)	28 (60.9)	
HER2/neu					
Positive	8 (53.3)	11 (29.7)	9 (19.1)	15 (32.6)	0.082
Negative	7 (46.7)	25 (70.3)	38 (80.9)	31 (67.4)	
Combined ER/PR status					
**+/+**	8 (53.3)	23 (62.2)	37 (78.7)	18 (39.1)	0.002*
**+/−**	0	2 (5.4)	3 (6.4)	10 (21.8)	
**−/+**	0	2 (5.4)	1 (2.1)	0	
**−/−**	7 (46.7)	10 (27.0)	6 (12.8)	18 (39.1)	
Tumor subtype					
Luminal A (ER+ and/or PR+, HER2-)	4 (26.7)	17 (46.0)	36 (76.6)	22(47.9)	0.006*
Luminal B (ER+ and/or PR+, HER2+)	4 (26.7)	10 (27.0)	5 (10.6)	7 (15.2)	
HER2-overexpression (ER-, PR-, HER2+)	4 (26.7)	2 (5.4)	3 (6.4)	10 (21.7)	
Triple negative (ER-, PR-, HER2-)	3 (19.9)	8 (21.6)	3 (6.4)	7 (15.2)	

Data are presented as the mean ± SD or number (%).

* *P* < 0.05 by chi-square test.

**Table 4:** Disease-free survival and overall survival rate of patients in different age groups and tumor stages.

Stage	Age groups	N	Disease-free survival						Overall survival					
			Mean	95% C.I.	Survival rate (%)			*P* value	Mean	95% C.I.	Survival rate (%)			*P* value
					3-yr	5-yr	10-yr				3-yr	5-yr	10-yr	
All	Very young (< 35)	15	93.9	65.2–122.6	73.3	66.7	38.9	0.003*	128.5	104.2–152.9	86.7	86.7	66.0	0.015*
	Young (36–40)	37	117.7	98.5–136.9	86.1	74.6	64.4		128.3	112.4–144.1	91.5	91.5	72.9	
	Premenopausal (41–50)	47	135.7	126.2–145.3	93.6	91.5	85.9		143.6	137.8–149.3	100.0	95.7	95.7	
	Postmenopausal (>50)	46	104.2	92.3–116.0	88.9	77.4	67.0		160.8	145.7–176.0	97.8	93.2	77.0	
I	Very young (< 35)	4	141.1	117.7–164.5	100.0	100.0	66.7	0.216	-	-	100.0	100.0	100.0	0.261
	Young (36–40)	10	116.6	82.1–151.0	88.9	77.8	55.6		141.0	121.1–160.9	100.0	100.0	88.9	
	Premenopausal (41–50)	25	136.5	123.9–149.0	96.0	92.0	87.4		144.1	137.1–151.1	100.0	96.0	96.0	
	Postmenopausal (>50)	17	104.1	85.4–122.8	94.1	81.1	65.5		121.1	111.9–130.2	100.0	100.0	75.5	
II	Very young (< 35)	6	62.9	30.3–95.5	66.7	50.0	16.7	0.004*	127.3	86.3–168.3	100.0	83.3	66.7	0.337
	Young (36–40)	21	133.9	114.5–153.3	90.5	85.7	85.7		135.9	118.5–153.4	90.5	90.5	84.0	
	Premenopausal (41–50)	13	114.4	101.8–126.9	92.3	92.3	79.1		-		100.0	100.0	100.0	
	Postmenopausal (>50)	16	98.5	78.0–118.9	81.3	75.0	68.8		162.7	137.6–187.7	100.0	93.8	79.3	
III	Very young (< 35)	5	85.6	33.8–137.3	60.0	60.0	40.0	0.003*	104.5	62.0–147.0	80.0	80.0	40.0	0.004*
	Young (36–40)	6	39.3	15.4–63.3	66.7	22.2	0.0		61.0	37.4–84.7	83.3	83.3	0.0	
	Premenopausal (41–50)	9	124.0	101.2–146.9	88.9	88.9	88.9		126.0	106.8–145.2	100.0	88.9	88.9	
	Postmenopausal (>50)	13	102.0	81.0–122.9	91.7	75.0	64.3		106.2	88.4–124.0	100.0	83.3	75.0	

*P < 0.05 by one-way ANOVA.

### Dysregulated miRNA between tumors and normal tissues

**Fig 4:**
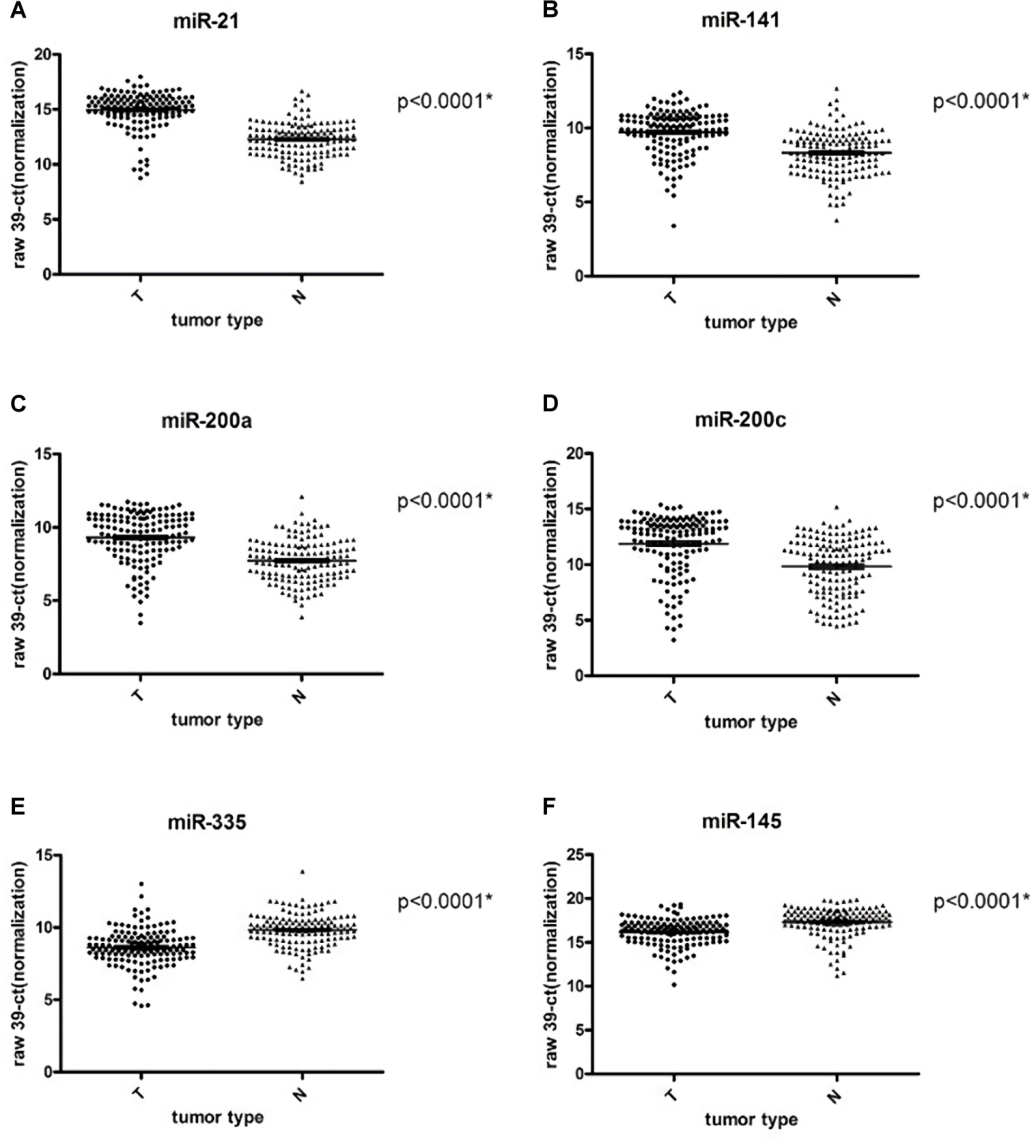
Dysregulated miRNAs between tumors and normal tissues. Up-regulated miRNAs: (A) miR-21, (B) miR-141, (C) miR-200a, (D) miR-200c; down-regulated miRNAs: (E) miR-335 and (F) miR-145 (tumor: n = 145; normal breast tissue: n = 140; *significant by the *t*-test).

When we compared the miRNAs expression in tumor (n = 145) and normal (n = 140) breast tissues, there were 6 miRNAs (miR-21, miR-141, miR-145, miR-200a, miR-200c, and miR-335) with significantly differential expression (Table 5). Among them, miR-145 and miR-335 were down-regulated in tumors, whereas miR-21, miR-200a, miR-200c, and miR-141 were up-regulated (Fig 4 and S2 Fig). Furthermore, a total of 38 miRNAs selected from our miRNA microarray data complete the qPCR analysis (Table 2), 31 miRNAs show the similar trend in two approaches. A high concordance rate (31/38, 89.5%) was observed between microarray analysis and qPCR validation.

**Table 5:** Differential expression of miRNAs in breast tumors.

MicroRNA	Mean expression^a^		FC^b^	*P* value^c^	*q* value^d^
	Tumor (n = 145)	Normal (n = 140)	T versus N		
Up-regulated					
miR-21	15.14	12.44	6.50	5.5E-38	1.7E-36
miR-200c	11.92	9.90	4.07	4.5E-13	2.7E-12
miR-200a	9.39	7.82	2.97	1.3E-15	1.3E-14
miR-141	9.70	8.33	2.59	1.6E-14	1.2E-13
Down-regulated					
miR-145	16.21	17.35	-2.20	1.4E-11	6.1E-11
miR-335	8.71	9.90	-2.28	2.9E-17	4.4E-16

The selection criteria of listed miRNAs as follows: |FC| > 2, *p* value < 0.05 and *q* value < 0.05.

^a^Mean expression levels expressed as 39-*C*_t_.

^b^FC, fold change values were log2 transformed.

^c^*P* values were calculated by *t*-test.

^d^*q* value, false discovery rate-adjusted *p* value.

### Specific miRNA expression in different age of onset groups

In each age group, we compared the miRNA expression between tumor and normal tissues respectively (Table 6). To identify specific miRNAs in different age groups, further compared the differentially expressed miRNA in tumor tissues between age groups (Table 6, in boldface). In addition to the 6 differential expression miRNAs between tumor and normal tissues (Table 6, highlighted with asterisks), there were 3 up-regulated (miR-96, miR-182, and miR-183) and 8 down-regulated (miR-10a, miR-10b, miR-125b, miR-127-3p, miR-130a, miR-143, miR-19, and miR-320) miRNAs detected in very young breast tumors (age < 35 y/o). For young breast tumors (36–40 y/o), 3 up-regulated (miR-30b, miR-30d, and miR-149) and 3 down-regulated (miR-487b, miR-548c-5p, and miR-181d) miRNAs were revealed. Only one specific down-regulated (miR-206) miRNA was observed in premenopausal breast tumors (41–50 y/o), and no unique miRNA was found in postmenopausal tumors (age > 50 y/o).

**Table 6:** Differential expression of miRNAs in specific age-onset groups.

	Age-onset groups			
	< 35 yr (FC)^a^	36–40 yr (FC)^a^	41–50 yr (FC)^a^	> 50 yr (FC)^a^
	(N = 15)	(N = 37)	(N = 47)	(N = 46)
	T versus N	T versus N	T versus N	T versus N
Up-regulated				
	**miR-96 (4.75)**	miR-21 (6.87)*	miR-21 (8.81)*	miR-21 (4.89)*
	**miR-183 (4.63)**	miR-200c (6.34)*	miR-200c (3.74)*	miR-200c (3.15)*
	miR-21 (4.30)*	miR-200a (4.00)*	miR-150 (2.66)	miR-200a (2.49)*
	**miR-182 (3.33)**	miR-141 (3.21)*	miR-200a (2.66)*	miR-141 (2.43)*
		**miR-149 (2.70)**	miR-30c (2.41)	
		miR-30c (2.47)	miR-141 (2.11)*	
		**miR-30d (2.11)**		
		miR-150 (2.11)		
		**miR-30b (2.05)**		
Down-regulated				
	**miR-320 (-2.04)**	**miR-181d (-2.05)**	**miR-206 (-2.06)**	miR-335 (-2.08)*
	**miR-10a (-2.2)**	**miR-487b (-2.12)**	miR-145 (-2.23)*	miR-145 (-2.26)*
	**miR-130 (-2.54)**	miR-335 (-2.22)*	miR-154 (-2.42)	
	**miR-127-3p (-3.01)**	**miR-548c-5p (-2.29)**	miR-335 (-2.52)*	
	**miR-143 (-3.47)**	miR-154 (-2.46)		
	**miR-10b (-3.86)**			
	**miR-125b (-5.77)**			
	miR-145 (-6.16)*			
	**miR-195 (-6.53)**			

MicroRNAs differentially expressed between the tumor and normal tissues in each group identified. The selection criteria of listed miRNAs as follows: |FC| > 2, *p* value by *t*-test < 0.05 and *q* value < 0.05.

^a^ FC, fold change values were log2 transformed.

* The differential expression miRNAs (miR-21, miR-200c, miR-200a, miR-141, miR-145 and miR-335) distinguish from comparison of breast tumor tissues and normal tissues.

Boldface indicates the unique differential expressed miRNAs in different onset age groups.

### Specific miRNA expression differences in different hormone receptor expression status and tumor subtypes

Investigate the differential expressed miRNAs in tumor with diverse hormone receptor expression status. The unique differential expressed miRNAs include that miR-375 was up-regulated in ER-positive tumors, and miR-17 was up-regulated in ER-negative tumors. The miR-9 and miR-205 were down-regulated in PR-positive and -negative tumors, respectively (Table 7, in boldface). In addition, miR-30c was up-regulated in HER2-negative breast tumors. In different tumor subtypes, miR-532-5p was up-regulated in triple-negative breast tumors. The miR-29c and miR-9 were up- and down- regulated in luminal B breast tumors, respectively. For HER2-overexpression breast tumors, 1 up-regulated (miR-155), and 4 down-regulated (miR-10b, miR-125b, miR-143 and miR-195) miRNAs were revealed (Table 8, in boldface).

**Table 7:** Differential expression of miRNAs in specific receptor expression status.

	Hormone receptor status					
	ER+ (FC)^a^	ER- (FC)^a^	PR+ (FC)^a^	PR- (FC)^a^	HER2+ (FC)^a^	HER2- (FC)^a^
	T versus N	T versus N	T versus N	T versus N	T versus N	T versus N
Up-regulated						
	miR-21 (7.05)*	miR-21 (5.29)*	miR-21 (7.50)*	miR-21 (4.96)*	miR-21 (6.25)*	miR-21 (6.61)*
	miR-200c (4.21)*	miR-200c (3.89)*	miR-200c (4.57)*	miR-200c (3.53)*	miR-200c (3.16)*	miR-200c (4.65)*
	miR-200a (3.24)*	miR-200a (2.51)*	miR-200a (3.32)*	miR-200a (2.54)*	miR-200a (2.96)*	miR-200a (3.01)*
	miR-141 (2.73)*	miR-141 (2.28)*	miR-141 (2.81)*	miR-141 (2.23)*	miR-141 (2.18)*	miR-141 (2.77)*
	miR-149 (2.41)	miR-532-5p (2.23)	miR-149 (2.36)	miR-532-5p (2.13)		miR-150 (2.11)
	**miR-375 (2.32)**	**miR-17 (2.16)**		miR-150 (2.12)		**miR-30c (2.09)**
		miR-150 (2.06)				
Down-regulated						
	miR-154 (-2.06)	miR-125b (-2.11)	**miR-9 (-2.18)**	miR-125b (-2.08)	miR-335 (-2.23)*	miR-145 (-2.04)*
	miR-335 (-2.15)*	**miR-143 (-2.18)**	miR-154 (-2.30)	**miR-205 (-2.09)**	miR-145 (-2.53)*	miR-154 (-2.13)
		miR-335 (-2.49)*	miR-335 (-2.31)*	miR-335 (-2.18)*		miR-335 (-2.28)*
		miR-145 (-2.96)*		miR-145 (-2.83)*		

MicroRNAs differentially expressed between the tumor and normal tissues in each group identified. The selection criteria of listed miRNAs as follows: |FC| > 2, *p* value by *t*-test < 0.05 and *q* value < 0.05.

^a^ FC, fold change values were log2 transformed.

* The differential expression miRNAs (miR-21, miR-200c, miR-200a, miR-141, miR-145 and miR-335) distinguish from comparison of breast tumor tissues and normal tissues.

Boldface indicates the unique differential expressed miRNAs in different hormone receptor status.

**Table 8:** Differential expression of miRNAs in specific tumor subtypes.

	Tumor subtypes			
	Luminal A (FC^a^)	Luminal B (FC^a^)	HER2-overexpression (FC^a^)	Triple negative (FC^a^)
	(ER+ and/or PR+, HER2-)	(ER+ and/or PR+, HER2+)	(ER-, PR-, HER2+)	(ER-, PR-, HER2-)
	T versus N	T versus N	T versus N	T versus N
Up-regulated				
	miR-21 (6.79)*	miR-21 (8.92)*	miR-21 (3.69)*	miR-21 (5.79)*
	miR-200c (4.38)*	miR-200c (4.03)*	**miR-155 (2.22)**	**miR-532-5p (3.12)**
	miR-200a (3.13)*	miR-200a (3.96)*		
	miR-141 (2.86)*	miR-149 (3.62)		
	miR-375 (2.11)	miR-141 (2.57)*		
	miR-149 (2.07)	miR-375 (2.38)		
		**miR-29c (2.23)**		
Down-regulated				
	miR-154 (-2.14)	**miR-9 (-2.03)**	**miR-10b (-2.12)**	
	miR-335 (-2.22)*	miR-154 (-2.05)	**miR-125b (-2.42)**	
		miR-145 (-2.09)*	**miR-143 (-2.54)**	
		miR-335 (-2.24)*	**miR-195 (-2.62)**	
			miR-145 (-3.55)*	

MicroRNAs differentially expressed between the tumor and normal tissues in each group identified. The selection criteria of listed miRNAs as follows: |FC| > 2, *p* value by *t*-test < 0.05 and *q* value < 0.05.

^a^ FC, fold change values were log2 transformed.

* The differential expression miRNAs (miR-21, miR-200c, miR-200a, miR-141, miR-145 and miR-335) distinguish from comparison of breast tumor tissues and normal tissues.

Boldface indicate the unique differential expressed miRNAs in different tumor subtypes.

## Discussion

A substantial difference exists between Asian and Western breast cancer regarding incidence and peak age [15]. Recent studies have demonstrated rising incidence and mortality rate of breast cancer in Asia. Invasive breast cancer incidence in the middle-age birth cohort is similar between Asian and Western populations. However, young generations in Taiwan are surpassing the historically high rates in the United States [16]. In this study, we found that young patients tended to have more advanced stage cancers with poor prognosis after 10 years follow-up. Clinical and molecular biological studies on this issue of age are important.

We designed a two-step approach, and the research subjects in the first step were young and included invasive in situ breast cancers. Young patients were expected to carry more genetic variants, so we analyzed the disease in situ to get further information. Histological lobular-type cancer was not included due to the rarity of these cases. The results showed that breast cancers in very young and young patients had different miRNA expression patterns compared with those in postmenopausal women. Similarly, other studies have noted that young breast cancer is a biologically distinct entity beyond the subtype distribution [17].

Some studies have revealed that miRNA dysregulation in young breast cancer is related to cell proliferation, motility and invasion pathways, which support their clinically aggressive courses and poor prognosis [18, 19]. In the present study, we found that three up-regulated miRNAs (miR-183, miR-96, and miR-182) in the very young breast cancer group were in an evolutionarily conserved miRNA cluster. Two of them, miR-96 and miR-182, have been reported to target a tumor-suppressor gene of the Forkhead Box O subfamily of transcription factors 1 (FOXO1). Down-regulation of FOXO1 by microRNAs in breast cancer cell lines may result in the transformation and maintenance of an oncogenic state of breast cells [20]. miR-183 has an oncogenic role through the regulation of the tumor-suppressor genes, EGR1 and PTEN [21]. Increases in miR-182 and miR-183 may contribute to early breast cancer development [22]. From the results, we assume that the evolutionarily conserved miRNA cluster is critical to initiate malignancy transformation of breast tissue in young adults.

miRNA expression profiling and validation in formalin-fixed paraffin-embedded samples has good reproducibility and biological accuracy. Different platforms of miRNA profiling technologies and pitfalls for qPCR might result in inconsistent results [23, 24].

We characterized the up-regulation of miR-21, miR-141, miR-200a, and miR-200c and down-regulation of miR-145 and miR-335, which were detected frequently in all patient groups. miR-21 functions as an oncogene targeting TPM1 and PDCD4 tumor suppressor genes. Up-regulation of miR-21 promotes breast cancer cell invasion and has been demonstrated to have diagnostic and prognostic potential in breast cancer [25, 26]. miR-200a, miR-200c and miR-141 are family members subject to epigenetic regulation in cancerous and normal tissues. Dynamic epigenetic regulation modulates epithelial and mesenchymal transitions in human tumorigenesis [27]. Loss of miR-200a expression is frequently observed in high-grade breast cancer [28]. miR-145 is hypothesized to be a tumor suppressor and has been reported to be down-regulated in breast cancer. It reduces breast cancer cell motility and invasiveness by targeting mucin 1 (MUC1) and fascin 1 [29]. miR-335 is located on the metastasis suppressor and tumor initiation suppressor locus 7q32.2. Genetic deletion of miR-335 is common in breast cancer and enriched in a metastatic setting [30].

The miR-10 family has been found to play an important role in developmental regulation by targeting Hox transcripts. Deregulation of miR-10 family members in different human cancers has been reported [31]. We also identified miR-10a and miR-10b as down-regulated in tumor cells breast cancers from very young patients. Decreased miR-10a expression, resulting in an increase in cell growth, has been reported in chronic myeloid leukemia and colorectal cancer studies [32, 33]. In breast cancer studies, an increase in the copy number of the miR-10a gene was found in primary tumor specimens and cell lines [34]. miR-10b is highly expressed in metastatic breast cancer cell lines, and the level of miR-10b expression is correlated with clinical progression [35]. Conversely, Gee et al. found lower miR-10b expression in early breast cancer patients without distant metastasis [36]. Our results imply that early stage population selection in this study and miR-10b are related to cancer progression and metastasis rather than initiation.

In patients with early recurrence, down regulation of five microRNAs in tumors has been reported: miR-149, miR-10a, miR-20b, miR-30a-3p and miR-342-5p [37]. We chose miR-149 and miR-10a as targets in our miRNA qPCR analysis based on our microarray data and literature review [38] respectively. From our analysis, in young breast cancer group, miR-10a was found to have similar down-regulation as reported but miR-149 was conflicting to that in the literature. miR-149 suppresses breast cancer cell invasion and metastasis by targeting GIT1 (G-protein-coupled receptor kinase-interacting protein 1). Low expression of miR-149 and high expression of GIT1 are associated with advanced stage breast cancer and lymph node metastasis [39]. Though miR-149 functions as a metastasis suppressor in most breast cancer studies, we found that miR-149 was up-regulated in the young breast cancer group. In other cancers, miR-149 inhibits cancer cell proliferation, such as in gastric cancer and astrocytomas [40, 41], but is associated with the development of nasopharyngeal carcinoma [42]. This discrepancy can be attributed to multitasking of microRNAs in regulating different downstream effectors. Different carcinogenic mechanisms of breast cancer between populations can also be one of the reasons.

Though 6 miRNAs dysregulation generally expressed in all age groups. MicroRNA dysregulation was less occurred in postmenopausal group than other age groups. Postmenopausal breast cancers may be caused by acquired genetic mutations resulting from environmental exposure such as diet, radiation, chemical, stress, or other risk factors. We speculate that miRNA dysregulation might alter the breast cancer occurrence in young adults.

Subtyping in breast cancer is important for treatment choices and prognosis prediction. Differential expression of miRNA in all subtypes of breast cancer has been thoroughly studied. Due to cancer heterogeneity, differences in miRNA abundance from the centers or edges of tumors, and different study methods are the probable reasons that characteristic miRNA expression profiles for each histologic subtypes lack. [43–46]. Our data presented higher prevalence of luminal A breast cancer in premenopausal (41–50 y/o) group than in postmenopausal group (> 50 y/o) and the subtype distribution was concordant with other study in Taiwan [2]. However, there still no specific miRNAs identified in luminal A subtype from our study (Table 8). Treatment strategies for triple-negative breast cancer were limited due to the lack of effective therapeutic targets. We discovered that miR-532-5p was up-regulated in the triple negative group. miR-532-5p inhibits cell proliferation and metastasis by targeting the CXCL2 oncogene in hepatocellular carcinoma [47]. However, it promotes cell growth, migration and invasion in gastric cancer cells and melanoma by down-regulating RUNX3 [48, 49]. The role of miR532-5p could be a probable biomarker for prognosis in triple-negative breast cancer.

Prognostic analyses in many reports have shown that after adjusting for race, histological grade, stage, hormone status and treatment, age was still an independent risk factor related to prognosis [5, 50]. Middle-aged patients have better overall survival than younger and elderly patients. Generally, this is most likely a result of early disease detection, less aggressive subtypes and better therapy tolerance [51].

In this study, miR-30c was up-regulated in the middle-aged group. miR-30c targets cytoskeleton network genes and regulates epithelial-to-mesenchymal transition [52]. It has also been reported to be an independent predictor of advanced breast cancer patients with tamoxifen treatment response and longer progression-free survival [53]. This miRNA alteration may be one of the reasons leading to better survival in middle-aged breast cancer patients. Furthermore, our results identified miR-30c up-regulation in the HER2-negtive group. As the luminal A group was the major proportion (78.2%) of HER2-negative tumors in our study. This implies that miR-30c up-regulation is related to good prognosis in breast cancer patients.

## Conclusion

In this study, we revealed differential miRNAs expression between age groups and subtypes of breast cancer in Taiwan. Some specific miRNAs limited to an individual group may be strongly associated with their clinical presentation. We also found that evolutionarily conserved miRNA clusters were up-regulated in very young adults, which may target some genes to initiate malignancy transformation. Young age was independently as a poor prognostic factor in long term follow-up. Less miRNA dysregulation was found in the postmenopausal group. In other words, miRNAs may play a particularly important role in tumor initiation in breast cancers in very young patients compared with other age groups.

Currently, miRNA is not only a predictor of chemoresistance and chemosensitivity but circulating miRNA could also be used as a biomarker for early cancer detection as well as subtype prediction. This study could provide information for risk prediction of early-onset breast cancer and research of ethnic diversity and therapeutic applications.

## Supporting information

S1 Fig Unsupervised hierarchical clustering of 54 differentially expressed miRNAs in 4 normal (blue) and 6 tumor (red) samples. The hierarchical clustering was performed using Squared Euclidean Distance as a distance measure and Ward's method for linkage analysis. MicroRNA levels were expressed as 39-*C*_t_ after global median normalization.
(TIF)

S2 Fig Unsupervised hierarchical clustering of 6 differentially expressed miRNAs in normal (red; n = 140) and tumor (blue; n = 145) samples. Hierarchical clustering was performed using Squared Euclidean Distance as a distance measure and Ward's method for linkage analysis.
(TIF)
