# Supplementary figures and images for: Differential microRNA expression in breast cancer with different onset age
Source: PLoS One. 2018 Jan 11;13(1):e0191195. doi: 10.1371/journal.pone.0191195 (PMC5764434; doi:10.1371/journal.pone.0191195)

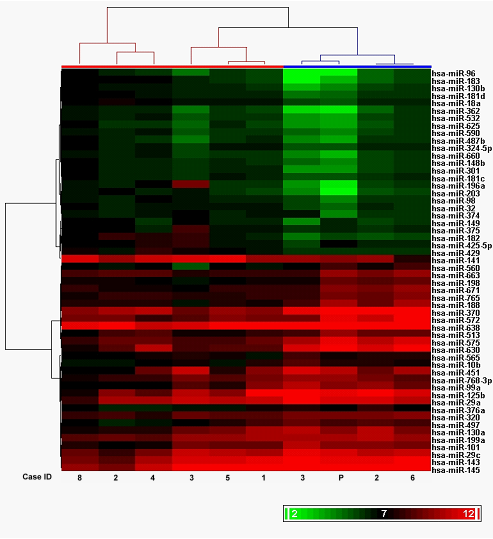

Supplement: S1 Fig — The hierarchical clustering was performed using Squared Euclidean Distance as a distance measure and Ward's method for linkage analysis. MicroRNA levels were expressed as 39-Ct after global median normalization. (TIF) [file pone.0191195.s001.tif]

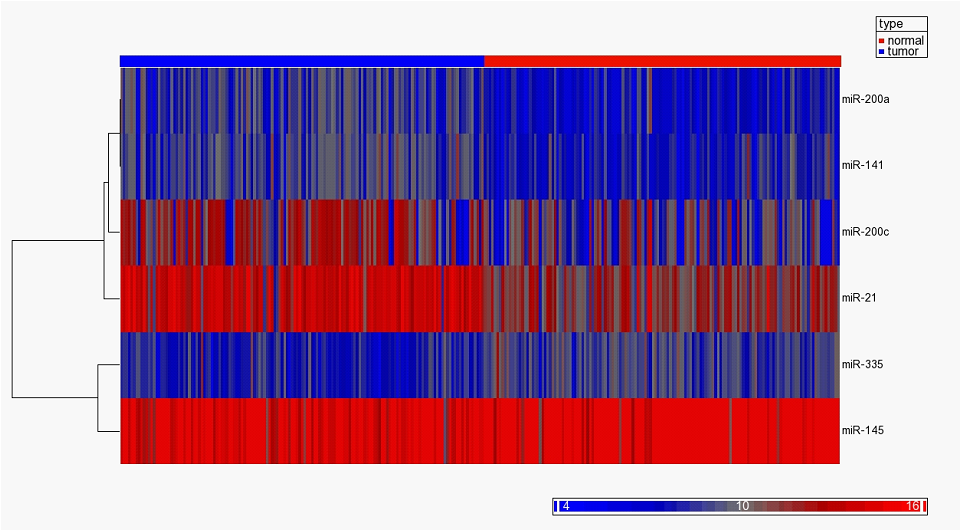

Supplement: S2 Fig — Hierarchical clustering was performed using Squared Euclidean Distance as a distance measure and Ward's method for linkage analysis. (TIF) [file pone.0191195.s002.tif]
